# Peripheral Microvascular Dysfunction in Children and Adults with Congenital Heart Disease: A Literature Review

**DOI:** 10.2174/011573403X278440240209064408

**Published:** 2024-02-21

**Authors:** Inne Vanreusel, Wendy Hens, Emeline Van Craenenbroeck, An Van Berendoncks, Vincent F.M. Segers

**Affiliations:** 1 Department of Cardiology, Antwerp University Hospital, 2650 Edegem, Belgium;; 2 Research Group Cardiovascular Diseases, GENCOR, University of Antwerp, 2610 Antwerp, Belgium;; 3 Cardiac Rehabilitation Centre, Antwerp University Hospital, 2650 Edegem, Belgium;; 4 Department of Rehabilitation Sciences and Physiotherapy, Faculty of Medicine and Health Sciences, MOVANT Research Group, University of Antwerp, 2000 Antwerp, Belgium

**Keywords:** Microvascular dys function, peripheral microvascular dys function, endothelial dys function, endothelium, vascular/physiopathology MeSH, congenital heart disease, heart defects, congenital MeSh

## Abstract

Although there is a continually growing number of patients with congenital heart disease (CHD) due to medical and surgical advances, these patients still have a poorer prognosis compared to healthy individuals of similar age. In patients with heart failure, microvascular dysfunction (MVD) has recently emerged as a crucial modulator of disease initiation and progression. Because of the substantial pathophysiological overlap between CHD and heart failure induced by other etiologies, MVD could be important in the pathophysiology of CHD as well. MVD is believed to be a systemic disease and may be manifested in several vascular beds. This review will focus on what is currently known about MVD in the peripheral vasculature in CHD. Therefore, a search on the direct assessment of the vasodilatory capacity of the peripheral microcirculation in patients with CHD was conducted in the PubMed database. Since there is little data available and the reported studies are also very heterogeneous, peripheral MVD in CHD is not sufficiently understood to date. Its exact extent and pathophysiological relevance remain to be elucidated in further research.

## 1 INTRODUCTION

Congenital heart disease (CHD) is one of the most frequently diagnosed congenital disorders, afflicting approximately 1% of live births worldwide [1]. Due to early management and improved surgical procedures, over 80% of CHD children now survive to adulthood, resulting in a continuously growing number of CHD patients [2-4]. CHDs form a complex and heterogeneous group of clinical entities with high morbidity and mortality [5]. Even after repair, patients with CHD have residual hemodynamic abnormalities, causing volume or pressure overload [2]. Development of heart failure (HF) is the main cause of morbidity and mortality in this population [6, 7]. Due to the substantial pathophysiological overlap between CHD and HF induced by other etiologies [4], the hypothesis of 'chronic HF induced by CHD' emerged, which is considered to apply to all CHD patients [2].

In HF, Microvascular Dysfunction (MVD) is known to be a key process [8]. In both HF with reduced and preserved ejection fraction, there is a systemic proinflammatory state with elevated oxidative stress and a reduced nitric oxide (NO) bioavailability, leading to endothelial dysfunction in large and small cardiac as well as extracardiac blood vessels and hence MVD [9-11]. Similarly, MVD could be important in the pathophysiology of CHDs as well. MVD is characterized by structural remodeling, rarefaction, reduced vasodilatory capacity, and platelet activation, leading to increased peripheral vascular resistance and a reduced blood supply to end organs [11]. Coronary MVD has recently been reviewed in CHD [4]. Therefore, this review will focus on MVD in the peripheral vasculature in CHD.

**Fig. (1):**
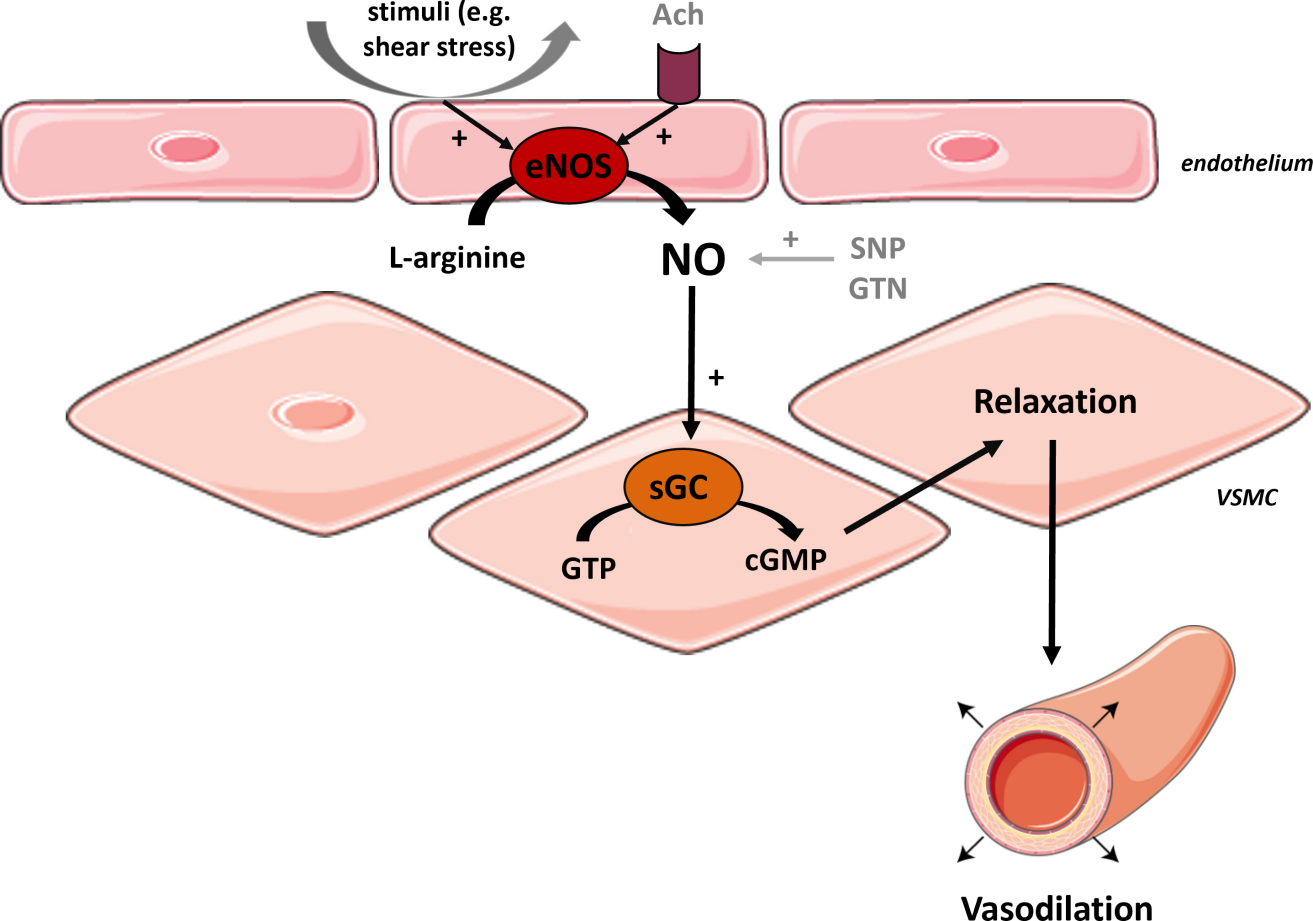
The mechanism underlying the vasodilatory response. The mechanism underlying the vasodilatory response is thought to be as follows: Under normal conditions, the activity of eNOS will be enhanced in response to a stimulus. This results in more NO production. Subsequently, NO diffuses into adjacent VSMCs and interacts with sGC, resulting in relaxation of VSMCs and consequent vasodilation [13]. The release of NO is mainly caused by shear stress, but can also be stimulated by agonists and vasoactive mediators (*e.g*., Ach) [10]. The concentration of NO can also be increased *via* exogenous administration of NO donors (*e.g*., SNP and GTN), which are prodrugs and rely on the generation of NO *in vivo* for their pharmacological activities [14]. • **Abbreviations:** Ach = acetylcholine, cGMP = cyclic guanosine monophosphate, eNOS = endothelial nitric oxide synthase, GTN = glyceryl trinitrate, GTP = guanosine triphosphate, sGC = soluble guanylyl cyclase, SNP = sodium nitroprusside, VSMC = vascular smooth muscle cell • *This figure was partly generated using Servier Medical Art, provided by Servier, licensed under a Creative Commons Attribution 3.0. unported license.*

Several pathophysiological mechanisms may underlie MVD [4]. In addition to endothelial cells, vascular smooth muscle cells (VSMCs), pericytes, and other cells in the microvessels are also crucial for the proper functioning of the small blood vessels [4]. There has been considerable interest in the assessment of endothelial function in cardiovascular disorders [12]. To this end, several methods have been developed that measure arterial vasodilation in response to intra-arterial infusion of endothelium-dependent (ED) vasodilators (*e.g*., acetylcholine) or reactive hyperemia [12]. Thus, these methods indirectly assess the ability of the endothelium to release endothelium-derived relaxing factors, among which Nitric Oxide (NO) is most studied. Because the vasodilatory response is affected by the endothelium and the responsiveness of VSMCs (Fig. **1**), the assessment of endothelial function presupposes a normal arterial response to NO [13]. Therefore, endothelium-independent (EID) vasodilation, namely the vascular response to exogenously administered nitroglycerine or nitroprusside, has classically been used as a method to differentiate impaired vasodilation caused by structural alterations or decreased responsiveness of VSMCs to NO from impaired vasodilation caused by endothelial dysfunction [13]. Nevertheless, there is evidence that not only ED vasodilation but also EID vasodilation is impaired in individuals with cardiovascular disease, indicating that also EID could be a prognostic marker of cardiovascular events [13]. Therefore, in this review, we included manuscripts on both ED and EID vasodilation in the peripheral microcirculation of CHD. In order to improve therapeutic interventions and preventive strategies, clinicians require a more comprehensive understanding of the underlying pathophysiological mechanisms in CHD [15].

The aim of this review is to gain more insight into peripheral microvascular function in patients born with CHD. Therefore, studies on the presence of peripheral MVD (PMD) in both children and adults with CHD have been reviewed. Furthermore, methods to assess peripheral microvascular function in CHD, factors associated with PMD, and therapies targeting PMD in CHD will also be discussed.

## 2 MATERIALS AND METHODS

The search was conducted in the PubMed database using the following terms: ((“Peripher*” OR “peripheral arterial disease” [MeSH Terms] OR “system*”) AND (“Microvascular” OR “Microcirculation” [MeSH] OR “Microvessels” [MeSH] OR “endothel*” OR “Endothelium, Vascular” [MeSH] AND (“Function” OR “dysfunction”)) AND (“Congenital heart disease” OR “Heart Defects, Congenital” [MeSH]. The in- and exclusion criteria outlined in Table **1** were employed to screen the identified papers through an assessment of their titles and abstracts. Only studies that compared the values of peripheral microvascular function tests in patients with CHD with a healthy control group or literature or across CHD groups were included. Additionally, the cited papers were scrutinized to discover any supplementary studies. This review ultimately incorporated a total of 20 papers.

## 3 RESULTS

PMD has been studied in heterogeneous patient populations consisting of multiple CHDs, but other studies focused on peripheral microvascular function in one particular type of CHD. Both cyanotic and acyanotic heart defects (cCHD and aCHD, respectively) are represented. The study populations mostly consisted of (young) adults, although children were also examined in some articles as well as patients with and without a history of surgical or percutaneous intervention. Notably, eight of the 20 studies were conducted in patients with repaired coarctation of the aorta (CoA).

### 3.1 Presence of PMD in CHD

Of the studies included in this review, 18 articles have investigated the presence of PMD in CHD. Therefore, the results of the CHD patients were usually compared with a healthy control group, matched for age, gender, and/or BMI. In two articles, however, only a comparison was made with other results from the literature. The results of these comparisons are shown in Table **2**, which illustrates that, to date, literature on the presence of PMD in CHD is conflicting: Ten studies conclude that PMD is present in CHD patients [2, 6, 16-23], while eight studies showed preserved peripheral vascular reactivity [24-31].

Importantly, the included studies used different measurement methods (Table **2**), which may or may not indicate abnormalities in different components of the peripheral vascular bed (*i.e.*, endothelium or VSMC).

### 3.2 Methods to assess PMD in CHD

**Fig. (2):**
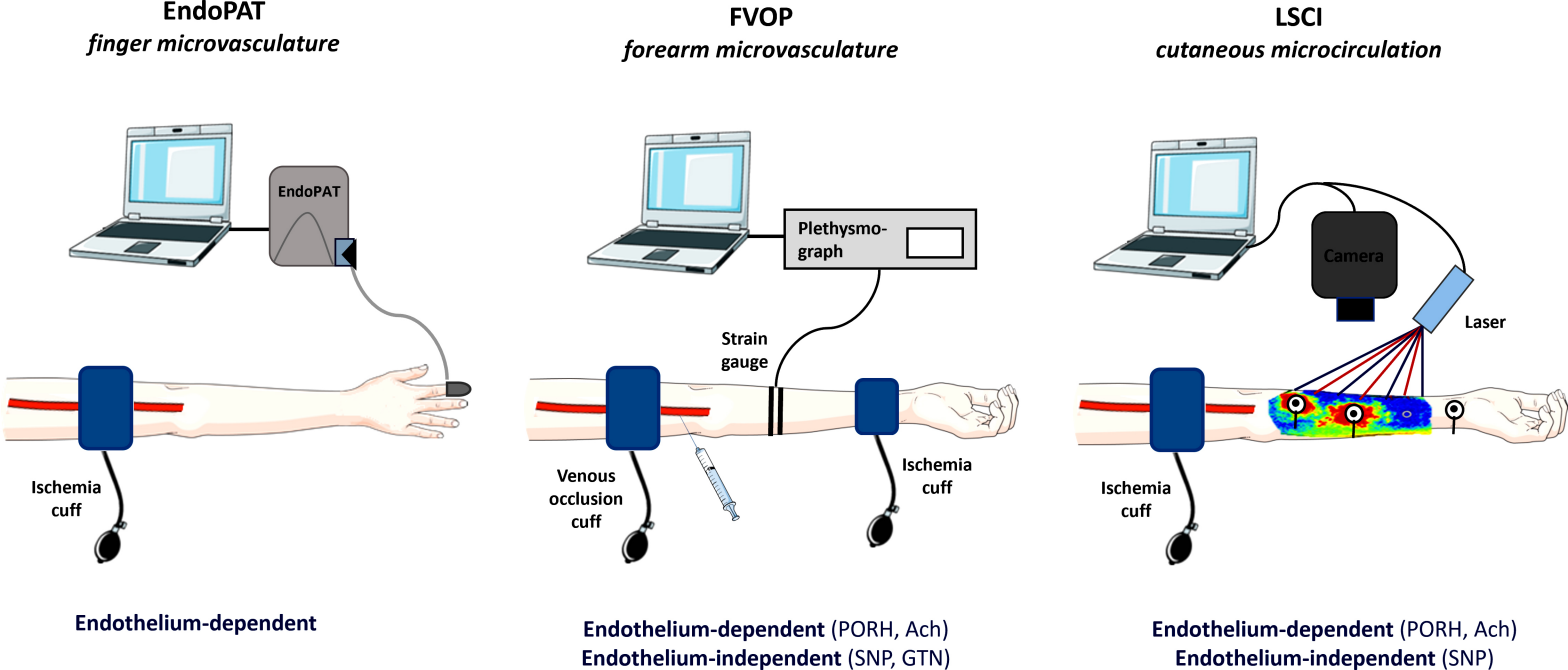
Different techniques to study PMD in CHD. **Abbreviations:** Ach = acetylcholine, FVOP = forearm venous occlusion plethysmography, GTN = glyceryl trinitrate, LSCI = laser speckle contrast imaging, PAT = peripheral arterial tonometry, PORH = post occlusion reactive hyperemia, SNP = sodium nitroprusside • *This figure was partly generated using Servier Medical Art, provided by Servier, licensed under a Creative Commons Attribution 3.0. unported license.*

Three different techniques have been used to study PMD in CHD: Finger plethysmography, forearm venous occlusion plethysmography (FVOP), and laser speckle contrast imaging (LSCI) (Fig. **2**).

#### 3.2.1 Finger Plethysmography by EndoPAT

Finger plethysmography is the most widely used technique to investigate PMD in CHD (Table **2**) and is used to assess flow-dependent endothelium-mediated peripheral artery function [28]. Using peripheral arterial tonometry (PAT), pneumatic finger probes capture beat-to-beat plethysmographic recordings of arterial pulse wave amplitudes [12]. An elevation in arterial blood volume at the fingertip leads to an increase in pulsatile arterial column changes, consequently influencing the measured arterial pulse wave amplitude [12]. A pressure cuff is employed to induce reactive (flow-mediated) hyperemia in one hand, while the non-occluded hand serves as a reference to normalize for any systemic changes in vascular tone during the test [12, 26]. Following the hyperemia induction, the occluded hand's reactive hyperemia index (RHI) is computed as the ratio between post-occlusion and pre-occlusion PAT signals normalized to the control hand [29].

The augmentation of pulse amplitude during reactive hyperemia is an intricate process, reflecting alterations in flow and digital microvessel dilatation. It is only partially dependent on NO [12, 16]. Abnormalities in vasodilator function in the peripheral finger, as measured by the EndoPAT device, have shown correlations with coronary microvascular function in patients with early atherosclerosis and have predictive value for cardiovascular events [12, 16]. The advantages of EndoPAT include feasibility, reproducibility, non-invasiveness, automated data analysis, and ease of use [16, 29].

#### 3.2.2 Plethysmography of the Forearm Circulation

Forearm venous occlusion plethysmography (FVOP) has also been used for the assessment of PMD in CHD (Table **2**). FVOP quantifies changes in forearm blood flow (FBF) using venous plethysmography in the forearm between two cuffs, before and after reactive hyperemia, and/or before and after infusion of vasoactive substances into a cannulated brachial artery [12, 32]. The underlying principle of FVOP is straightforward: When venous drainage from the arm is impeded but arterial inflow remains unaffected, blood will enter but not exit the forearm [32]. Consequently, there is an increase in forearm volume proportional to arterial blood inflow [32]. Assuming a constant perfusion pressure (arterial blood pressure), alterations in flow reflect changes in VSMC tone in resistance vessels [33]. Changes in forearm volume result in a corresponding alteration in arm circumference and strain gauge length, which are measured by a plethysmograph [32].

To briefly interrupt venous return from the forearm, a cuff is inflated around the upper arm to a pressure above venous but below diastolic pressure, typically 40 mmHg [32]. During measurements, circulation to the hands is excluded by rapidly inflating a smaller cuff around the wrist to suprasystolic pressure [32]. After obtaining baseline values, the impact of reactive hyperemia and/or vasoactive substances is studied in one arm. FBF measurements, on the other arm, serve as a control for potential systemic effects [21].

For inducing reactive hyperemia, the cuff at the distal part of the forearm is inflated above systolic pressure and deflated after five minutes to induce reactive (flow-mediated) hyperemia [22]. The forearm vasodilatory response to reactive hyperemia is defined as the percentage change of FBF from baseline to the maximum FBF during reactive hyperemia [22]. This post-occlusion reactive hyperemia (PORH) is widely used as an index of endothelial function in human forearm circulation [34]. Additionally, vasoactive molecules, hormones, or drugs can also be administered, enabling the quantification of ED and EID vasodilation, respectively, in a dose-dependent manner. Again, the results are expressed as the ratio of the changes in flow measured in both arms [12]. Vasoactive substances used in CHD include acetylcholine (Ach), which induces ED vasodilatation, glyceryl trinitrate (GTN) and sodium nitroprusside (SNP) responsible for EID vasodilation, and NO synthase (NOS) blocker N^G^-monomethyl-L-arginine (L-NMMA) to study NO bioavailability. Although this technique is suitable for measuring differences in FBF of different stimuli or inhibitors in one patient, its utility for comparisons between groups or serial studies in the same patient is limited [12]. Additionally, the semi-invasive nature of the technique, involving arterial puncture, remains a notable disadvantage [12].

#### 3.2.3 Laser Speckle Contrast Imaging

Laser speckle contrast imaging (LSCI) is a non-invasive technique that measures changes in cutaneous microcirculatory flow during pharmacological and physiological provocations [6]. LCSI has been used to evaluate PMD in CHD in two studies [6, 31], in which skin iontophoresis of ED (Ach) and EID (SNP) vasodilators and post occlusion reactive hyperemia (PORH) have been applied (Table **2**).

Microvascular flow recordings are conducted on randomly chosen skin sites on the ventral surface of the forearm [35]. Two drug delivery electrodes are glued to the skin for iontophoresis of Ach and SNP, respectively. Three areas (circular regions of interest) are then measured, with two of these areas located inside the electrode area (for measurement of Ach and SNP) and the third area located adjacent to the electrode to measure PORH [6]. Following a five‐minute baseline recording of microvascular blood flow, iontophoresis with Ach or SNP is performed with anode currents (Ach) or cathode currents (SNP) of increasing intensities [6]. A neutral electrode is used for current dispersion. For the PORH test, brachial artery occlusion is induced using a pneumatic cuff inflated at suprasystolic pressure for three minutes [6]. Upon cuff release, maximum cutaneous blood flow is measured [6, 35].

In the two studies on CHD, the results were reported differently. Weismann *et al*. [31] selected the mean perfusion over the baseline cycle length and the cycle with the highest mean response for analyses. In contrast, Marino *et al*. [6] divided the measurements of skin blood flow by the mean arterial pressure to yield the cutaneous vascular conductance (CVC) expressed in arbitrary perfusion units (APU)/mmHg. Subsequently, response amplitudes were expressed as the peak CVC minus the baseline CVC [6].

### 3.3 PMD as a Marker of the Systemic Nature of CHD?

**Fig. (3):**
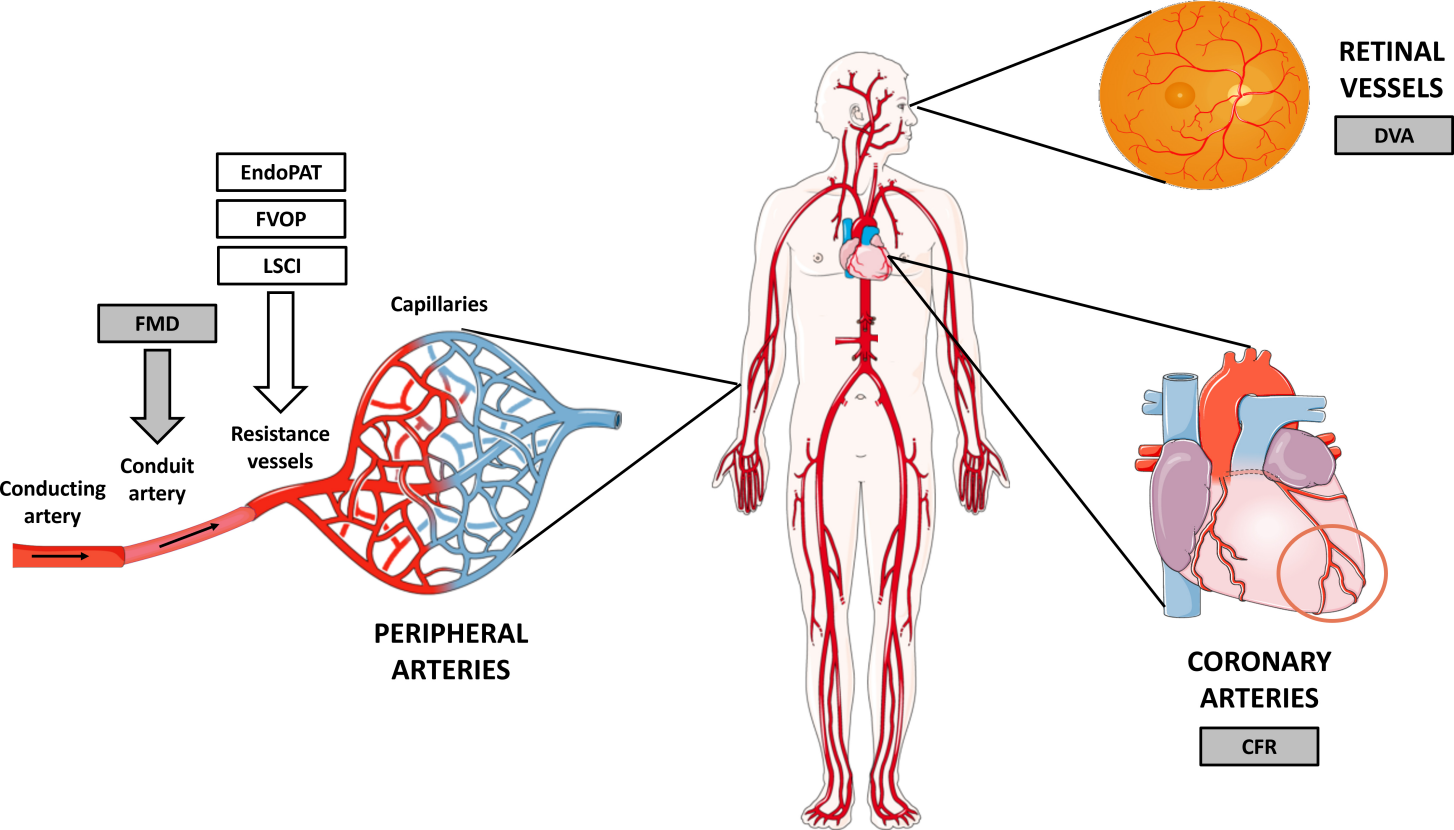
Different techniques to assess endothelial function in different vascular beds. One may consider FMD on the one hand and EndoPAT, FVOP and LSCI on the other as complementary, since these techniques assess endothelial function in distinct segments of the arterial tree. FMD offers insights into arterial conduit vessels, while RHI and FBF, as assessed by EndoPAT, FVOP and LSCI, provide information about endothelial function in the resistance vessels [32]. Utilizing these methods collectively allows for a comprehensive evaluation of endothelial function across different parts of the arterial vasculature. • **Abbreviations:** CFR = coronary flow reserve, DVA = dynamic vessel analysis (flicker response), FMD = flow-mediated dilatation, FVOP = forearm venous occlusion plethysmography, LSCI = laser speckle contrast imaging, PAT peripheral arterial tonometry •**Source:** *This figure was partly generated using Servier Medical Art, provided by Servier, licensed under a Creative Commons Attribution 3.0. unported license, and based on [*37].

Endothelial dysfunction is commonly perceived as a systemic disorder affecting coronary arteries and the peripheral vascular bed, encompassing both conduit arteries and small resistance vessels in the extremities (Fig. **3**) [36]. While cardiovascular risk factors are linked to endothelial dysfunction across various arterial beds, it is important to recognize the differences in reactivity between conduit and resistance arteries [12]. In the conduit arteries, reduced NO release in response to stimuli plays a central role in the pathophysiology of endothelial dysfunction [12]. On the other hand, in resistance arteries, ED vasodilation is also influenced by metabolic and other factors in addition to NO [12]. Consequently, pharmacological tests that induce NO release may not fully reflect the physiological adaption of endothelial function in the microvasculature in response to exercise or ischemia [12]. Furthermore, endothelial function assessments vary in sensitivity to different risk factors. Flow-mediated dilation (FMD) impairment, a systemic indicator of endothelial dysfunction in conduit arteries, is notably sensitive to traditional risk factors such as age and hypertension [12, 25]. In contrast, EndoPAT demonstrates greater sensitivity to metabolic risk factors, particularly body mass index and diabetes mellitus [12, 25].

Concerning CHD, only three articles investigated endothelial function in other vascular beds in addition to the peripheral small resistance vessels, and they reported different results [17, 24, 25]. In a heterogeneous group of cCHD, widespread endothelium dysfunction appears to exist, as these patients showed impaired FMD in the brachial artery and attenuated flicker response in the small arteries and veins of the retina [24]. In the distal arterial bed of the fingertip, however, there was a non-significant trend toward endothelial dysfunction [24]. In patients with repaired CoA, endothelial dysfunction is more pronounced in conduit arteries than in resistance arteries [25], but in adolescent and young adult Fontan survivors, endothelial dysfunction is more pronounced in resistance arteries (reduced RHI) than in conduit arteries (FMD values similar to values in healthy controls) [17].

The cCHD patient group of Cordina *et al*. [24] is older (mean age 41 ± 3 years) than the other two groups (22.0 ± 6.9 [25] and 13.9 ± 4.1 years [17]), and because age is an important risk factor for decreased FMD, this may have contributed to the lower FMD values. The body mass index (BMI) of the patients was reported to be significantly lower than that of the control group [24], which could potentially have an impact on the results related to the RHI. The authors also acknowledge that complex blood changes at various levels of the vascular bed may contribute to variations in the impact of erythrocytosis. Subsequently, the amount of shear stress may differ significantly between large vessels and the microvasculature [24].

It is possible that compensatory erythrocytosis in cyanosis leads to chronically elevated shear stress in large vessels, with the microvasculature relatively less affected [24, 38]. However, these results are contradicted by the findings of Goldstein *et al*. [17] in Fontan patients and therefore controversial. In patients with repaired CoA, published by Nozaki *et al*. [25], 28% of the patients had arterial hypertension requiring antihypertensive treatment, which may have contributed to the reduced FMD, and, although hemoglobin A1c (HbA1c) was higher in the CoA group than in the control group, only one patient had diabetes mellitus, and there was no significant difference between the two groups in markers of lipid metabolism.

None of the studies investigated coronary microvascular function in addition to the peripheral small resistance vessels. Nevertheless, two studies investigating CHD [39, 40] examined coronary microvascular function and peripheral endothelial function using FMD. These studies revealed that there was no statistically significant correlation between FMD and coronary flow reserve in CHD. Consequently, there is no established link between coronary and peripheral endothelial function at the microvascular level in patients with CHD [4].

### 3.4 Pathophysiology of PMD in CHD

The existing literature provides a foundation for speculative consideration of the involved pathophysiological mechanisms [6]. PMD in CHD is likely to arise from a complex interplay of various factors [24]. Potential contributing factors to PMD in CHD (or the other way around) that have been studied in the included articles are discussed in this section.

#### 3.4.1 Patient Characteristics

Although only little studied, age [19], gender [16, 19], BMI [16], HbA1c [25], and family history of cardiovascular risk factors [16] were not related to peripheral microvascular function. However, in treated CoA patients, age at treatment was significantly associated with the EndoPAT index [28].

#### 3.4.2 Blood Pressure, use of Antihypertensive Drugs, and Sympathetic Activity

The relationship between peripheral microvascular function and blood pressure (BP) has been investigated several times in CHD, more specifically in patients with repaired CoA and patients with Fontan circulation. At first, there seems to be no relationship between peripheral microvascular function and BP (and left ventricular mass index) [25] in patients with repaired CoA [25, 29], and PMD has also been identified in normotensive patients with successfully repaired CoA [22]. However, in contrast to these studies, Lee *et al*. [23] did find impaired RHI in CoA patients with elevated clinic BP compared to those with normal BP. Nevertheless, the same association was not observed on 24-hour BP monitoring nor with the PAT ratio.

At the same time, muscle sympathetic nerve activity (MSNA) was also notably higher in the CoA patients who exhibited elevated BP in the clinic, though again not on 24-hour BP monitoring (indicative of white coat hypertension) [23]. Excessive sympathetic nervous system activity has been demonstrated to compromise endothelial function in both healthy individuals and those with chronic HF [18]. However, there was no correlation between endothelial function and MSNA in the repaired CoA patients [23]. Lambert *et al*. [18] demonstrated an increased sympathetic nerve activation in Fontan patients, in whom MSNA activation may serve as a necessary adaptation to maintain systemic blood perfusion and organ perfusion. Unfortunately, no correlation between RHI and MSNA was studied in this study.

Interestingly, in Fontan patients, no significant differences were observed in the PAT ratio related to the use of angiotensin-converting-enzyme (ACE) inhibitors [16]. The absence of an effect of ACE inhibition on vascular function is a noteworthy finding, particularly given that a substantial portion of Fontan subjects are on ACE inhibitor therapy, and this medication has demonstrated improved endothelial function in other populations [16]. Similarly, although PMD was present in the CoA patients, they did not show plasma renin levels different from controls [23].

#### 3.4.3 Cyanosis and Hypoxia

Cyanosis can result from Eisenmenger physiology, low pulmonary blood flow, or a right-to-left shunt without Eisenmenger physiology [24]. In patients with cCHD, chronic hypoxemia, and subsequent erythrocytosis bring about significant changes in blood vessel structure and function [21, 38]. Compensatory erythrocytosis causes a substantial, chronic increase in whole blood viscosity and wall shear stress [21, 38]. Notably, acute and chronic changes in shear stress exert distinct effects on the vasculature [38]: While an acute rise in blood viscosity and shear stress can induce an increase in basal NO release, leading to subsequent vasodilation, a chronic elevation in blood viscosity and shear stress, as observed in compensatory erythrocytosis, may have a more intricate effect resulting in vascular remodeling, characterized by an increased vessel diameter. This remodeling aims to normalize shear stress on the vessel wall [38, 41]. This chronic elevation in blood viscosity and shear stress could also potentially lead to a blunted response to mechanical stimuli [38, 42]. Besides shear stress, oxygen is a well-established local regulator of vascular tone. Acute hypoxia induces NO secretion and vasodilatation, whereas chronic hypoxia induces vasoconstriction also *via* an ED mechanism. Another important consideration in erythrocytosis is the possibility that hemoglobin is scavenging NO [38]. Indeed, the reduced response to the NOS inhibitor L-NMMA in cCHD implies a diminished basal bioavailability of NO [21]. Furthermore, the potent vasoconstrictor endothelin-1 (ET-1) is also secreted in response to heightened vascular shear stress [24].

Arguing against this hypothesis are the findings that circulating ET-1 levels are not elevated in a heterogenous group of cCHD [21] nor in Fontan patients [18]. Additionally, the patients with cCHD and healthy subjects did not exhibit significant differences in vascular response to the blockade of endogenous ET-1 [21]. In contrast, in another group of cCHD, levels of ET-1 and asymmetric dimethylarginine (ADMA) were found to be increased, consistent with an imbalance between vasodilating and vasoconstricting factors, but these patients did not demonstrate PMD measured in the fingertip [24].

Overall, most studies examining peripheral microvascular function in cCHD indeed report PMD [16-18, 21]. A significant correlation was even found between oxygen saturation and the maximal response to Ach in cCHD [21]. Nevertheless, PMD was not present in all cyanotic defects [24, 26] and no study has actually compared peripheral microvascular function between a group of cyanotic and acyanotic heart defects. In addition, Cordina *et al*. [24] could also not confirm a significant correlation with oxygen saturation. Finally, since PMD is observed in aCHD in addition to cCHD, this indicates that cyanosis and hypoxemia are at least not the only factors responsible for PMD in CHD.

#### 3.4.4 Pulmonary Blood Flow and Pulmonary Arterial Hypertension

Endothelial dysfunction in the pulmonary vascular bed stands as a significant underlying mechanism of pulmonary arterial hypertension (PAH) [26]. In theory, factors contributing to endothelial dysfunction within the pulmonary circulation could potentially affect the systemic circulation if released in sufficient amounts [21]. Nevertheless, the precise nature and extent of the involvement of peripheral or systemic vasculature in PAH remain unclear [26]. Patients with PAH exhibit chronically impaired production of vasodilators, such as prostacyclin and NO, alongside an overexpression of vasoconstrictors, including ET-1 [26]. However, the results regarding ET-1 levels are contradictory [18, 21, 24]. Moreover, studies on the involvement of the peripheral vasculature in CHD with altered pulmonary blood flow (PBF) and PAH are scarce.

It appears that both decreased and increased PBF can be associated with PMD. In Fontan patients, a frequently proposed explanation for the observed endothelial dysfunction is a decreased pulsatility in the pulmonary circulation leading to a decreased shear stress–mediated release of NO [18]; indeed, all three papers studying Fontan circulations report PMD [16-18]. On the other hand, in congenital cardiac shunt lesions (CSL), it is not a reduced, but rather the increased PBF that might be responsible for the PMD. The underlying mechanism could be a decrease in cardiac output and basal limb perfusion associated with the increased PBF caused by the cardiac shunt [20]. This lower blood flow in peripheral tissues can lead to a decrease vascular wall shear stress and endothelial dysfunction [20]. Indeed, Nakamura *et al*. [20] showed that FBF response induced by Ach was significantly lower in patients with CSL. Interestingly, vascular responses in CSL patients with severe PAH seemed to differ from those with mild PAH since SNP-induced response was attenuated only in the former group. It has been proposed that prolonged endothelial dysfunction, particularly impairment of the NO system, can trigger vascular smooth muscle and structural alterations [20]. Consequently, endothelial dysfunction may manifest in patients with mild CSL, but as CSL progresses to a more advanced stage, endothelial dysfunction may contribute to VSMC and matrix changes, ultimately leading to generalized peripheral vasodilator dysfunction [20]. In fact, impaired vasodilator response to Ach and SNP in the upper limbs have been demonstrated as a sensitive and specific marker to identify patients with advanced pulmonary circulatory abnormalities, such as Eisenmenger's syndrome in adult patients with CSL, across a wide range of pulmonary arterial pressures [20]. Peled *et al*. [26] also investigated patients with Eisenmenger syndrome due to CSL, but surprisingly, no difference in PAT ratio from the healthy controls could be observed in the Eisenmenger syndrome group. In addition, Oeschlin *et al*. [21] did not find differences in their study results between cCHD patients with and without PAH. It should be noted, however, that the number of patients without PAH was small. Finally, in a heterogeneous group of CHD with altered PBF, endothelial function in the fingertip was also not significantly impaired [24], and capillary wedge pressure was not related to blood flow response in chronic HF secondary to CHD [19].

Together, these findings suggest that changes in PBF and PAH may partly contribute to PMD but alone are insufficient to explain PMD in CHD.

#### 3.4.5 Disease Severity

The existing literature regarding the relationship between peripheral microvascular function and disease severity presents conflicting evidence. The oldest study included in this review has shown that both ED and EID vasodilation are impaired in symptomatic patients with chronic HF secondary to CHD [19]. Notably, despite the absence of a relationship between cardiac index and blood flow response, vascular dysfunction worsened with the clinical progression of HF symptoms, as indicated by the New York Heart Association (NYHA) functional class [19]. Moreover, as previously mentioned, impaired upper limb vasodilator responses (to Ach and SNP) have been identified as a useful marker for identifying patients with advanced pulmonary circulatory abnormalities such as Eisenmenger's syndrome in patients with CSL [20]. These findings underscore the importance of HF severity in vascular dysfunction [19]. However, althorugh Goldstein *et al*. [16] found a weak correlation between PAT ratio and peak oxygen consumption, peak work, and quality-of-life in their overall study population (Fontan patients and healthy controls), these correlations were not significant when the groups were analyzed independently. PAT-RHI also did not correlate with measures of exercise performance in another Fontan population [17]. In a group of adults with cCHD, no correlation between RHI and functional status (6-minute walking distance) could be established [24], and the distribution of a heterogeneous group of cyanotic and acyanotic CHD patients according to Weber's classification also did not result in a significant difference in peripheral microvascular function [6]. Interestingly, in patients with PAH, the Eisenmenger patients were the only group with a normal PAT ratio, albeit with lower oxygen saturation and higher pulmonary pressure [26]. The impaired hyperemic response of the overall patient group exhibited a significant correlation with disease severity. This correlation was determined through various measures, including the NYHA classification, pulmonary pressure, 6-minute walking distance, and oxygen desaturation on effort [26]. Exclusion of the Eisenmenger syndrome group from the analysis yielded stronger correlations [26]. Finally, although PMD was present, no differences in N-terminal pro–B-type natriuretic peptide (NT-proBNP) levels were reported between repaired CoA patients and healthy controls [2]. This absence of differences in NT-proBNP levels may be attributed to several factors. These include a normal average NYHA, the lack of left ventricular pressure overload, and left ventricular function and dimension within a normal range [2].

#### 3.4.6 Inflammation, Oxidative Stress, and Endothelial Repair

PMD may arise from dysfunctional VSMCs and/or impaired endothelial cells (Fig. **1**). Endothelial cells have multiple functions, such as acting as a barrier between circulating blood and underlying cells like VSMCs and cardiomyocytes. Additionally, endothelial cells actively secrete various small molecules, peptides, and proteins [43]. This secretion of paracrine factors enables endothelial cells to influence the function of the underlying VSMCs and cardiomyocytes [43]. The balance of endothelial injury and recovery affects endothelial function [29]. Various tissues, including the immune system, vascular wall, and myocardium, produce proinflammatory cytokines, such as tumor necrosis factor alpha (TNFα), monocyte chemoattractant protein-1 (MCP-1), interleukin-1 beta (IL-1β), IL-6 and IL-8. These proinflammatory cytokines impair endothelial function by reducing NO synthesis [22]. They also increase the production of adhesion molecules (*e.g*., soluble vascular cell adhesion molecule-1 (sVCAM-1), soluble intercellular adhesion molecule-1 (sICAM-1) and E-selectin in vascular endothelium and stimulate the synthesis of acute-phase proteins (high-sensitivity C-reactive protein (hs-CRP) and fibrinogen) in the liver [22, 29, 34]. Additionally, besides the reduced synthesis of NO, endothelial dysfunction can result from decreased availability of the amino acid substrate for NO production, L-arginine, or increased inactivation of NO by free radicals [44].

**Fig. (4):**
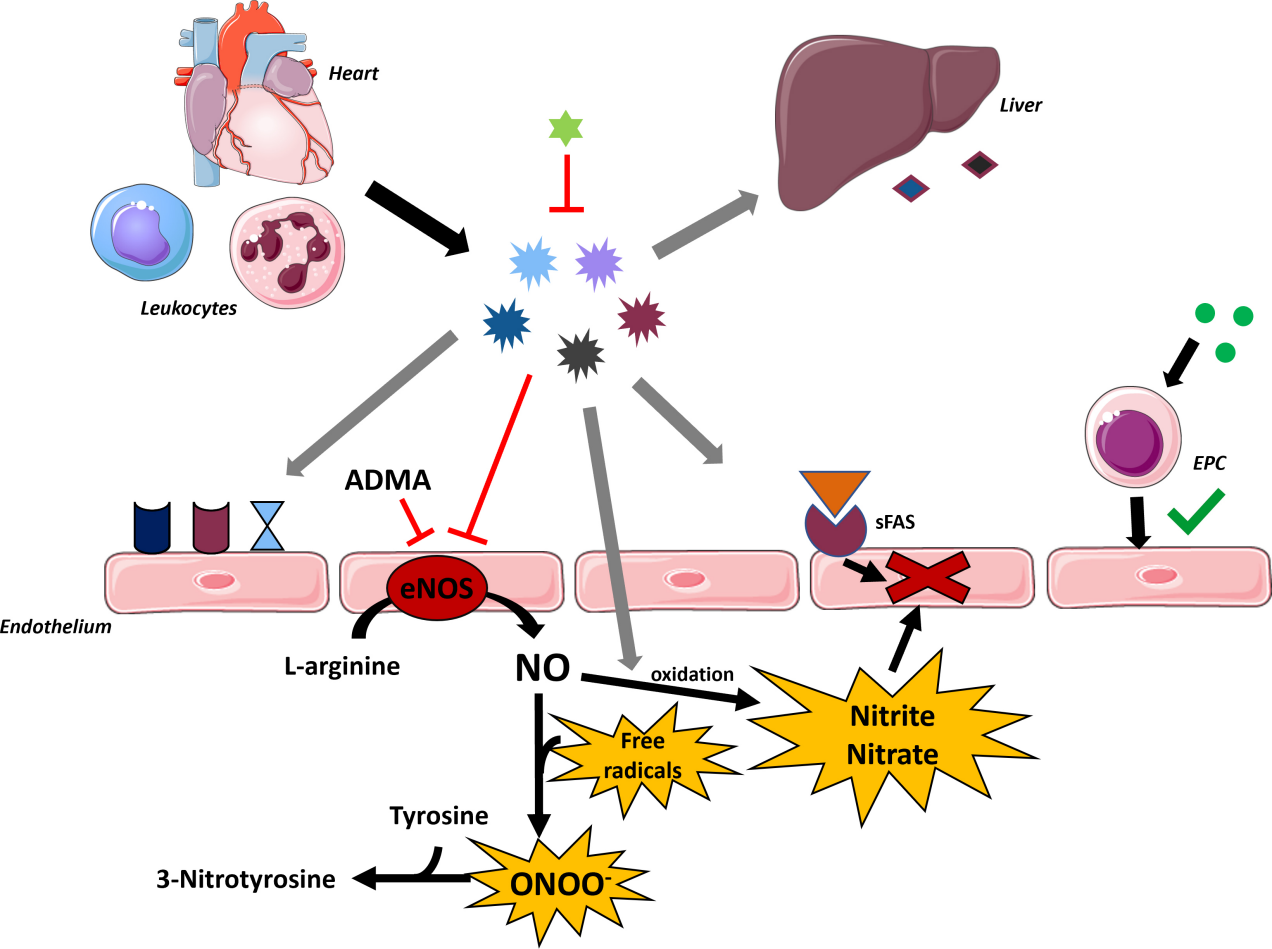
Markers of inflammation, oxidative stress and endothelial repair studied in papers investigating peripheral microvascular function in CHD.

In several of the studies included in this review, markers of inflammation, oxidative stress, and endothelial repair have been studied; all but one [24] of these studies involved CoA patients. Comparing plasma levels of these markers in CHD and healthy controls led to conflicting results (Fig. **4**, Table **3**). However, it is striking that CHD patients with PMD showed higher plasma levels of inflammatory markers [2, 22], while the levels of inflammatory markers were the same in healthy controls and CHD patients without PMD [24, 25, 29]. Thus, this suggests a potential association between increased inflammation and endothelial dysfunction [22]. Also, with the exception of high-sensitivity C-reactive protein (hs-CRP) levels, which were lower in the balloon dilatation group, other blood biomarkers were similar across treatment groups among patients with CoA. This observation is consistent with the finding that treatment modality (surgery, balloon dilatation or stent) in CoA was not associated with differences in endothelial function [28]. Moreover, increased levels of the soluble ligand of protein FAS (sFAS-L) were observed in CHD patients with PMD, implying the involvement of apoptosis of cardiomyocytes and endothelial cells [2].

Regarding endothelial repair in CHD patients, two key factors studied are circulating endothelial progenitor cells (EPCs) and vascular endothelial growth factor (VEGF). VEGF serves as a potent angiogenic factor that stimulates the mobilization of EPCs from the bone marrow to replace injured endothelial cells. Reduced levels of VEGF are implicated in the development of endothelial dysfunction [29]. VEGF and EPC levels were investigated in only two studies, both involving CHD patients without PMD [24, 29]. Not surprisingly, the numbers of EPCs and VEGF levels did not significantly differ between patients with repaired CoA and controls [29]. However, in adults with cCHD, levels of EPCs were significantly reduced [24]. It is possible that chronic cyanosis in cCHD patients inhibits EPC generation - either directly or through associated hemorheological changes - and, in this manner, contributes to the abnormal vascular responses in the brachial artery and the vessels of the retina [24].

Finally, two studies in repaired CoA patients also tested markers of oxidative stress and NO bioavailability and metabolism. Compared to healthy controls, there were no differences in nitrite/nitrate and 3-nitrotyrosine levels, consistent with the absence of PMD in these CHD patients [24, 28]. However, results regarding ADMA levels were conflicting [24, 28].

#### 3.4.7 History of Surgical or Percutaneous Intervention

It is unclear whether a surgical or percutaneous intervention affects peripheral microvascular function in CHD. Firstly, in patients with a history of surgical or percutaneous intervention, both preserved and impaired peripheral microvascular function have been observed (Table **2**). Secondly, the surgical history is not specified in all articles, and if specified, no comparison has been made of microvascular function before and after the procedure. As a result, it is not possible to state with certainty whether an intervention has a negative impact on peripheral microvascular function or whether peripheral vascular function in CHD patients with PMD will recover after surgical or percutaneous correction. Nevertheless, a few studies compared peripheral microvascular function between patients with and without prior intervention or between different types of interventions. For example, in patients with repaired CoA, RHI exhibited comparability between patients who underwent angioplasty and those who underwent surgery and these values were not influenced by the number of interventions [25]. Another study also found that peripheral microvascular function in CoA patients was similar across the different treatment modalities (surgery, balloon dilatation, or stenting) [28]. The results remained unchanged even after adjusting for potential confounding factors, like age at treatment [28]. Radke *et al.* [29] also found that the timing of surgical repair seems not to influence peripheral microvascular function in repaired CoA patients. A final study examining the effect of an intervention was in patients with a bicuspid aortic valve (BAV), where findings on microvascular function were preserved when excluding BAV patients who had previously undergone aortic valve replacement [30]. However, it should be noted that in all these studies, there was no PMD in the CHD patients or healthy controls, which may have biased the conclusions on the effect of interventions.

### 3.5 Treatment Options Targeting PMD in CHD

Only two articles have studied therapies targeting PMD in CHD, namely vitamin C [44] and ramipril [34].

Antioxidants have been demonstrated to prevent the inactivation of NO and preserve ED vasodilation, with vitamin C specifically shown to improve endothelial function in patients with conditions such as hypertension or chronic HF [44]. In high-performing asymptomatic Fontan patients, a four-week oral administration of vitamin C did not lead to improvements in vascular function as assessed by digital PAT [44]. However, among subjects with abnormal vascular function at baseline, vitamin C administration showed a higher frequency of normalization in both the EndoPAT index and PAT ratio compared with placebo [44]. Although this difference was not significant, it may suggest that antioxidant therapy might benefit a subset of Fontan patients with abnormal vascular function [44]. However, further investigations involving a broader spectrum of the Fontan population and extended duration are essential to determine the role of antioxidant therapy in the long-term treatment of Fontan patients conclusively [44].

In another study, treatment with ramipril (5mg/day) for 4 weeks led to an improvement in endothelial function, as observed through measurement of PORH but not through FBF response to nitroglycerin, in normotensive patients with successful CoA repair (SCR), independent of BP lowering [34]. Besides their antihypertensive effect, ACE inhibitors are recognized for their anti-inflammatory properties, enhancement of NO bioavailability, and direct interference with mechanisms of atherogenesis [34]. Indeed, ramipril also reduced the expression of IL-6, which is a proatherogenic inflammatory cytokine, adhesion molecule sVCAM-1, and serum soluble CD40 ligand (sCD40L) in this population. However, the treatment course did not affect serum levels of CRP and IL-1β [34]. These findings suggest that renin-angiotensin system blockade may reduce overall cardiovascular risk in normotensive SCR patients [34]. In Fontan patients, no significant difference was found in the PAT ratio related to the use of an ACE inhibitor [16].

## FUTURE PERSPECTIVES AND LIMITATIONS

Although MVD seems to play a crucial role in the pathophysiology of different forms of HF (4), current knowledge about PMD in CHD is limited. Its exact extent and pathophysiological relevance remain to be elucidated. Because of the substantial pathophysiological overlap between CHD and HF, PMD can be expected to be important in CHD as well and, therefore, may enable the identification of CHD with an unfavorable prognosis. Moreover, it could be a therapeutic target and have the potential to increase both the survival and quality of life in CHD patients.

In this review, the literature currently available on peripheral microvascular (dys) function in CHD is summarized, which as of now remains inconclusive. Because there are many different types of CHD, a subgroup analysis per individual CHD seems more useful than a general analysis in which all types of CHD are combined. However, the number of studies focusing on one particular type of heart defect or reporting results per specific CHD is limited. In addition, the included studies have a small sample size, and the reported results are often contradictory. These are the major reasons why it is very difficult to draw solid conclusions. Although some associations were investigated, the underlying mechanism of abnormal peripheral microvascular function was often unknown. Further research on this topic is needed. Understanding the mechanisms underlying PMD in CHD could potentially pave the way for therapeutic advancements, enhancing exercise capacity and ultimately improving the survival of these patients [38].

MVD, in general, can arise from various pathophysiological mechanisms, and different CHDs may involve distinct mechanisms of PMD. In this review, both ED and EID vasodilation were assessed in the peripheral microcirculation of CHD. Endothelial dysfunction is characterized by impaired ED vasodilatation resulting from diminished NO bioactivity and endothelial activation. The latter involves the release of cytokines and chemokines, creating a pro-inflammatory and pro-thrombotic environment [38, 45]. In studies assessing vasodilatation in the peripheral microcirculation in CHD, markers of inflammation, oxidative stress, and endothelial repair have also been studied several times. Although no correlations with PMD were investigated, plasma levels of these markers were compared in CHD to healthy controls. As mentioned earlier, the results of these comparisons are conflicting. Studies exclusively focused on other pathologic conditions of endothelial dysfunction (such as changes in anticoagulant and anti-inflammatory properties as well as impaired modulation of vascular growth and remodeling) or those that solely conducted indirect assessments of ED vasodilatation (*e.g*., plasma levels of NO, L-arginine, …) were excluded from this review. Additionally, the vasodilatory response, being a ratio, is influenced not only by conditions affecting maximal blood flow but also by basal flow velocities, further complicating the comparison of results [4].

The study populations in the included articles predominantly comprised (young) adults, with some articles also examining children. It has to be noted that the consistency of the three measurement techniques in children at different ages and adults has not been evaluated. Through the years, techniques to assess endothelial function have been used to monitor cardiovascular disease in adults [46]. In children, on the other hand, studies on endothelial function with, for example, EndoPAT devices are sparse [46]. There is, therefore, no cut-off value for the RHI in pediatric patients [47]. In addition, there is also an age-related difference in skin blood flow [48]. For this reason, we have chosen to include only studies comparing the values of peripheral microvascular function tests with those of a healthy control group (or literature).

Endothelial dysfunction is commonly considered a systemic phenomenon, affecting both resistance and conduit vessels in the coronary circulation and the forearm [32]. However, in CHD, this has been studied only to a limited extent. As we have discussed, the results concerning the involvement of both resistance and conduit vessels in CHD are contradictory [17, 24, 25]. Since this review focuses on the peripheral microcirculation and, thus, resistance vessels, articles that only examined conduit arteries were excluded. In fact, EndoPAT may provide practical advantages over brachial artery reactivity testing, including less extensive training requirements and a faster learning curve (16). Moreover, currently, there is no established link between coronary and peripheral microvascular function in CHD patients, similar to other forms of HF [4].

Furthermore, despite the high prevalence of PMD linked to adverse clinical outcomes in individuals with different cardiovascular diseases [12], the current understanding of the significance and long-term prognostic impact of diminished ED and/or EID vasodilatation in CHD remains unclear. Due to the substantial pathophysiological overlap between CHD and HF, PMD can also be expected to be important in CHD. However, it is conceivable that CHD may develop an adaptive compensatory mechanism distinct from patients with HF [6]. Notably, in contrast to the conventional HF model, where the left ventricle is typically implicated, impairment predominantly arises in the right cavities and in the pulmonary circulation in most CHDs [6]. Additionally, unlike CHD, HF primarily afflicts older individuals with associated comorbidities, which are known to be linked with endothelial dysfunction [6].

Up to now, only two author groups have studied whether there are treatment options targeting PMD in CHD [34, 44]. In normotensive patients with SCR, a four-week treatment course with ramipril led to an improvement in endothelial function [34] and although not statistically significant [44], administration of vitamin C could be promising to improve peripheral microvascular function in CHD. To substantiate these findings, studies are still needed in different CHD populations and for a longer duration. In addition, numerous other pharmacologic agents have been demonstrated to enhance endothelial function in other diseases and should be considered for testing in CHD [24]. Moreover, given that endothelial dysfunction may contribute to effort intolerance in individuals with HF and CHD, aerobic training could potentially serve as an effective therapeutic option, as evidenced in the case of HF [6].

A meta-analysis has not been performed due to the high heterogeneity (in types of CHD and patient characteristics, in measurement techniques to assess peripheral microvascular function in CHD, and in comparison with a control population or existing literature) between the studies. Unfortunately, we cannot rule out that the enormous heterogeneity of the literature causes a bias in our conclusions. Therefore, in the future, when further research allows more homogeneous groups and measurement methods to be combined, it would be useful to perform a meta-analysis to make a more objective statement about the presence or absence of PMD in CHD.

## CONCLUSION

MVD is believed to be crucial in the pathophysiology of various forms of HF. Because of the substantial pathophysiological overlap between CHD and other forms of HF, PMD can also be expected to be important in CHD. However, since there is little data available and the reported studies are also very heterogeneous, peripheral MVD in CHD is not sufficiently understood to date. Its exact extent and pathophysiological relevance remain to be elucidated in further research. Endothelial dysfunction is commonly considered a systemic phenomenon, but this has been studied only to a limited extent in CHD. Several pharmacologic agents have demonstrated efficacy in improving endothelial function in various cardiovascular diseases. In CHD, however, only vitamin C and ramipril have been studied, but these drugs appear to be promising. Further research in this area is necessary, as gaining insights into the mechanisms underlying PMD in CHD holds the potential for therapeutic advancements, enhancing exercise capacity and ultimately improving the survival of these patients.

## Figures and Tables

**Table 1:** In- and exclusion criteria for papers in this review

**Category**	**Inclusion Criteria**	**Exclusion Criteria**
**Design**	Analytic studies (comparison with a healthy control group or with literature, or comparison across CHD groups)	No full-text available in English
**Patient population **	Children, adolescents, and adults with CHD	Syndromic patients Research performed in animals
**Assessment method**	Direct assessment of ED and EID vasodilatation in the peripheral microcirculation (resistance vessels)	Studies concerning exclusively vascular function in the larger blood vessels (*e.g*., FMD for assessment of the conduit arteries) Studies exclusively assessing coronary, pulmonary, or central (retinal and cerebral) blood vessels Only indirect assessments of ED vasodilatation (*e.g*., plasma levels of NO, L-arginine, …) Assessment of exclusively other pathologic conditions of endothelial dysfunction (*e.g*., altered anticoagulant and anti-inflammatory properties and impaired modulation of vascular growth and remodeling)

**Abbreviations:** CHD = congenital heart disease, ED = endothelium-dependent, EID = endothelium-independent, FMD = flow-mediated dilation, NO = nitric oxide.

**Table 2:** Summary of studies on the presence of PMD in patients with CHD (grouped by pathology).

**Pathology**	**Age Group**	**Surgery (S) /** **Percutaneous (P)**	**Controls**	**Method**	**Conclusion** **(↔ Controls)**	**Results/** **Response to** **(↔ Controls)**	**References**
**CHD (NS) + HF**	Adults	NS	Healthy	FVOP	PMD present	• Ach ↓ • SNP ↓	[19]
**cCHD & aCHD: heterogenous group**	Adults	80% (77% S, 3% P)	Healthy	LSCI	PMD present	• Ach = • SNP ↓ • PORH =	[6]
**cCHD: heterogenous group**	Adults	Table 1 from [21]	Healthy	FVOP	PMD present	• Ach ↓ • SNP = • L-NMMA ↓	[21]
		±50% (S)	Healthy	EndoPAT	No PMD	RHI =	[24]
**Single ventricle (Fontan)**	Children - adults	Yes (S)	Healthy	EndoPAT	PMD present	RHI ↓	[16]
			Literature	EndoPAT	PMD present	RHI ↓	[17]
	Adults	Yes (S)	Healthy	EndoPAT	PMD present	RHI ↓	[18]
**TGA after ASO**	Children - adults	Yes (S)	Healthy	EndoPAT	No PMD	RHI =	[27]
**EMS due to CSL**	Adults	NS	Healthy	EndoPAT	No PMD	RHI =	[26]
**CSL with low and high PVR**	Adults	NS	Healthy	FVOP	PMD present	• Ach ↓ in both CSL with low and high PVR • SNP = in low PVR, ↓ in high PVR	[20]
**After CoA repair**	Children - adults	Yes (84% S, 5% P, 11% unknown, 7% P reintervention)^1^	Healthy	EndoPAT	No PMD	RHI =	[31]
				LSCI		• Ach = • SNP =	
		Yes (S or P, 33% multiple procedures: Table 1 from [25]	Healthy	EndoPAT	No PMD	RHI =	[25]
			Surgery ↔ angioplasty			Surgery = angioplasty	
		Yes (37% S, 63% P)^3^	Literature	EndoPAT	No PMD	RHI =	[28]
			Surgery ↔ BD ↔ stent			Surgery = BD = stent	
			BAV ↔ no BAV			BAV = no BAV	
		Yes (47% S, 32% P, 21% S+P)^4^	Healthy	FVOP	PMD present	PORH ↓	[2]
	Adults	Yes (S)	Healthy	EndoPAT	No PMD	RHI =	[29]
		Yes (S)	Healthy	FVOP	PMD present	• PORH ↓ • GTN =	[22]
		Yes (S)^5^	Healthy	EndoPAT	PMD present	RHI ↓	[23]
**BAV**	Children - adults	44% (S)	Healthy	EndoPAT	No PMD	RHI =	[30]

**Abbrevations:** Ach = acetylcholine, ASD = atrial septal defect, ASO = arterial switch operation, BAV = bicuspid aortic valve, BD = balloon dilation, CHD = congenital heart disease, CoA = coarctation of the aorta, CSL = cardiac shunt lesions, EMS = Eisenmenger syndrome, FVOP = forearm venous occlusion plethysmography, GTN = glyceryl trinitrate, HF = heart failure, L-NMMA = N^G^-monomethyl-L-arginine, LSCI = laser speckle contrast imaging, NS = not specified, PORH = post-occlusion reactive hyperemia, RHI = reactive hyperemia index, SNP = sodium nitroprusside, TGA = transposition of the great arteries, VSD = ventricular septal defect ^1^ 68% of the patients had a BAV. 13% of the patients had a mean left ventricular outflow tract gradient in the moderate range (20-30mmHg) and 38% had mild aortic insufficiency. 25% underwent procedures for associated CHD (ASD, VSD, (sub)aortic stenosis, mitral valve surgery), ^2^ 39% had a BAV, 17% had mild aortic valve stenosis, 44% had aortic valve regurgitation, ^3^ BAV was present in 71% of the surgery group, in 45% of the BD group and in 50% of the stent group, ^4^ BAV was present in 37% of the patients, ^5^ associated CHD and procedures: Table **2** from [23].

**Table 3:** Legend to figure 4.

**Marker**	**↑**	**=**	**Marker**	**↑**	**=**	**Marker**	**↑**	**↓**	**=**
IL-1β 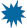	[22]^+^	[28]	sICAM-1 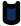	[22]^+^, [2]^+^	[29]	(hs)-CRP 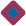	-	[28]^a^	[22]^+^,[25], [24], [28]
IL-6 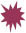	-	[22]^+^, [2]^+^,[29]	sVCAM-1 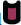	[22]^+^, [2]^+^	[28, 29]	Fibrinogen 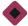		-	[22]^+^
IL-8 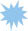	-	[29]	E-selectin 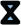	[22]^+^, [2]^+^	[24]	sFAS-L 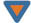	[2]^+^	-	-
TNFα 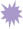	-	[2]^+^	3-nitrotyrosine	-	[28]	EPC	-	[24]	[29]
MCP-1 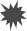	[29]	-	Nitrite/nitrate	[24]	[28]	VEGF 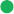	-	-	[29]
IL-10 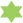	(2)^+^	-	ADMA	-	[29]	-	-	-	-

**Note:** ↑/↓/= higher/lower/equal levels in CHD compared to healthy controls, (or between treatment groups [28]) ^+^ = study indicating PMD, ^a^ = only balloon dilation group, ^b^ = surgery and stent group • **Abbreviations:** ADMA = asymmetric dimethylarginine, eNOS = endothelial nitric oxide synthase, EPC = endothelial progenitor cell, (hs)-CRP = (high)-sensitivity C-reactive protein, IL = interleukin, MCP-1 = monocyte chemoattractant protein-1, NO = nitric oxide, ONOO- = peroxinitrite, sFAS-L = soluble ligand of protein FAS, sICAM-1 = soluble intercellular adhesion molecule-1, sVCAM-1 = soluble vascular cell adhesion molecule-1, TNFα = tumor necrosis factor alpha, VEGF = vascular endothelial growth factor • *This figure was partly generated using Servier Medical Art, provided by Servier, licensed under a Creative Commons Attribution 3.0. unported license and based on [**37**].*

## LIST OF ABBREVIATIONS

(a/c)CHD: (Acyanotic/Cyanotic) Congenital Heart Disease
(e)NOS: (Endothelial) Nitric Oxide Synthase
ACE: Angiotensin-Converting-Enzyme
Ach: Acetylcholine
ADMA: Asymmetric Dimethylarginine
APU: Arbitrary Perfusion Units
ASD: Atrial Septal Defect
ASO: Arterial Switch Operation
BAV: Bicuspid Aortic Valve
BD: Balloon Dilation
BMI: Body Mass Index
BP: Blood Pressure
cGMP: Cyclic Guanosine Monophosphate
CoA: Coarctation of the Aorta
CSL: Cardiac Shunt Lesions
CVC: Cutaneous Vascular Conductance
DVA: Dynamic Vessel Analysis
ED: Endothelium-Dependent
EID: Endothelium-Independent
EMS: Eisenmenger Syndrome
EPC: Endothelial Progenitor Cell
ET-1: Endothelin-1
FBF: Forearm Blood Flow
FMD: Flow-Mediated Dilation
FVOP: Forearm Venous Occlusion Plethysmography
GTN: Glyceryl Trinitrate
GTP: Guanosine Triphosphate
HbA1c: Hemoglobin A1c
HF: Heart Failure
hs-CRP: High-Sensitivity C-Reactive Protein
IL: Interleukin
L-NMMA: N^G^-Monomethyl-L-Arginine
LSCI: Laser Speckle Contrast Imaging
MCP-1: Monocyte Chemoattractant Protein-1
MSNA: Muscle Sympathetic Nerve Activity
MVD: Microvascular Dysfunction
NO: Nitric Oxide
NT-proBNP: N-Terminal Pro–B-type Natriuretic Peptide
NYHA: New York Heart Association
PAH: Pulmonary Arterial Hypertension
PAT: Peripheral Arterial Tonometry
PBF: Pulmonary Blood Flow
PMD: Peripheral Microvascular Dysfunction
PORH: Post-Occlusion Reactive Hyperemia
RHI: Reactive Hyperemia Index
SCR: Successful CoA Repair
sFAS-L: Soluble Ligand of Protein FAS
sGC: Soluble Guanylyl Cyclase
sICAM-1: Soluble Intercellular Adhesion Molecule-1
SNP: Sodium Nitroprusside
sVCAM-1: Soluble Vascular Cell Adhesion Molecule-1
TGA: Transposition of the Great Arteries
TNFα: Tumor Necrosis Factor-Alpha
VEGF: Vascular Endothelial Growth Factor
VSD: Ventricular Septal Defect
VSMC: Vascular Smooth Muscle Cell
